# Long non-coding RNA HOTAIRM1-1 silencing in cartilage tissue induces osteoarthritis through microRNA-125b

**DOI:** 10.3892/etm.2022.11327

**Published:** 2022-04-19

**Authors:** Wen-Bin Liu, Gui-Shi Li, Peng Shen, Ya-Nan Li, Fu-Jiang Zhang

Exp Ther Med 22:933, 2021; DOI: 10.3892/etm.2021.10365

Following the publication of the above paper, the authors wish to publish a Corrigendum to account for a necessary change in the authorship on the paper. Essentially, the second author, Qi-Jin Feng, requested that they be removed from the paper due to disagreements over the interpretation of certain of the experiments. All the authors agree to the indicated change of authorship on this paper, and agree with the publication of this corrigendum. Therefore, the revised authors’ names on this paper, together with their affiliations, are as follows:

WEN-BIN LIU^1*^, GUI-SHI LI^2^, PENG SHEN^3^, YA-NAN LI^4^ and FU-JIANG ZHANG^1^

^1^Department of Joint Surgery, Tianjin Hospital, Tianjin 300211; ^2^Department of Joint Surgery, Yuhuangding Hospital, Yantai, Shandong 264000; ^3^Department of Rheumatology and Immunology, Tianjin First Center Hospital, Tianjin 300192; ^4^Department of Orthopedics, Tianjin Dongli Hospital, Tianjin 300300, P.R. China

The authors are grateful to the Editor of *Experimental and Therapuetic Medicine* for allowing them the opportunity to publish this Corrigendum, and apologize for any inconvenience caused.

